# Gas Bacillus Infection

**Published:** 1915-11

**Authors:** 


**Gas Bacillus Infection.** G. M. Barrett, San Francisco, Cal. State Jour. of Med., July, reviews the literature and reports personal experience. The Bacillus Welchii grows on nearly all media in the absence of oxygen, produces butyric acid and gas consisting of 64.3% H, 27.6% carbon dioxid, 8.1% N. Owing to the excess of H tne gas burns with a pale blue flame. In the presence of oxygen, it does not produce gas and its virulence is slight. Cramp found 9 cases in 5802 traumatisms at Bellevue. He collected 187 cases from the literature, of which 21 were post-operative. He gives the following:

Conclusions: 1. B. Welchii is probably not found in open wound as a pure infection but always associated with other organisms of more or less pathogenicity. 2. The B. Welchii may be a harmful invader of the human organism, but usually only in cases of local or systemic devitalizations, or when acting as a symbiote. 3. The proteolytic effect of B. Welchii is much augmented by the presence in the tissues, at the same time, of B. Coli, as this latter bacillus ferments out the sugar, allowing a much more rapid disintegration of the protein, with a consequent increase in the absorption of the resulting putrefactive products, and so a greater toxemia. 4. When present in a wound, this organism causes a peculiar sickening odor, a reddish watery discharge, and under certain conditions, gas and crepitation; and the requisites for a cure are to reduce the edema which makes for increased virulence by reduction of oxygen and production of acid, both tending to an increased autolysis of tissue; by free deep incisions and the use of a solution of sol. chlor. 1% and sod. citr. 2%, or peroxide of hydrogen; and restore the blood and oxygen supply by removal of sutures, if any, exposed to the air, application of oxygen gas, or peroxide of hydrogen, though it is doubtful if these agents are effective except on the surface. The direct injection of oxygen into the tissues has been recommended, but I know of no series of cases proving its worth and reliability. Of course, interference with, or destruction of the blood supply may be so complete it cannot be restored. 5. Amputation, in most cases, does not materially enhance the patient's chances of recovery, where an extremity is involved, but rather militates against it. This is not to be taken to mean that if amputation is indicated from other causes it should be delayed. 6. Plaster casts should not be employed as a first dressing, if at all in those fracture cases with extensive trauma, crushing and known introduction of dirt or other infective material. Anerobic and aerobic cultures from the wound and from the blood should be taken in all such severe traumatic cases. 7. Death is usually not due to the B. Welchii alone, but is more often the result of shock or sepsis, or both, though the gas bacillus may sometimes aid by symbiosis.

